# Low Levels of the Herbicide Atrazine Alter Sex Ratios and Reduce Metamorphic Success in *Rana pipiens* Tadpoles Raised in Outdoor Mesocosms

**DOI:** 10.1289/ehp.0901418

**Published:** 2009-11-19

**Authors:** Valérie S. Langlois, Amanda C. Carew, Bruce D. Pauli, Michael G. Wade, Gerard M. Cooke, Vance L. Trudeau

**Affiliations:** 1 Centre for Advanced Research in Environmental Genomics (CAREG), Department of Biology, University of Ottawa, Ottawa, Ontario, Canada; 2 National Wildlife Research Centre; 3 Environmental Health Science and Research Bureau and; 4 Toxicology Research Division, Health Canada, Ottawa, Ontario, Canada

**Keywords:** 5β-reductase, amphibians, aromatase, atrazine, enzyme activity, feminization, gonadal development, metamorphosis, Northern leopard frog, real-time RT-PCR

## Abstract

**Background:**

There are conflicting reports regarding the effects of atrazine (ATZ) on amphibian development. Therefore, further studies are needed to examine the potential mechanisms of action of ATZ in amphibians.

**Objectives:**

Our aim in this study was to determine whether low concentrations of ATZ affect gonadal development and metamorphosis in the Northern leopard frog, *Rana pipiens*.

**Methods:**

Tadpoles were exposed in outdoor mesocosms to nominal concentrations of 0.1 and 1.8 μg/L of formulated ATZ from Gosner stage 27 (G27) to metamorphic climax (G42). Exposure to 17α-ethinylestradiol (EE_2_; 1.5 μg/L) provided a positive control for induction of testicular oocytes in males. Endocrine-related gene expression and gonadal histopathology were examined at G42 and in a subset of premetamorphic G34 tadpoles that failed to metamorphose.

**Results:**

Gonadal gross morphology revealed that the 1.8-μg/L ATZ treatment produced 20% more females compared with the control. Histologic analysis revealed that 22% of EE_2_-treated males had testicular oocytes, whereas none were observed in any animals from the control or either ATZ groups. ATZ increased brain estrogen receptor α mRNA to 2.5 times that of the control at premetamorphosis and altered liver levels of 5β-reductase activity at metamorphosis. In contrast, brain aromatase mRNA level and activity did not change. ATZ treatments significantly reduced metamorphic success (number of animals reaching metamorphosis) without affecting body weight, snout–vent length, or age at metamorphosis. Gene expression analysis indicated that ATZ decreased the expression of deiodinase type 3 in the tail at premetamorphosis.

**Conclusions:**

Our study indicates that exposure to low concentrations of ATZ in experimental mesocosms alters gonadal differentiation and metamorphosis in developing *R. pipiens.*

There is controversial evidence that the widely used herbicide atrazine (ATZ) may alter gonadal development by affecting gonadal steroidogenesis through alteration of aromatase activity (Hayes et al. 2002b). Aromatase (cyp19) is a cytochrome P450 enzyme that converts testosterone into estradiol (Lephart 1996) and androstenedione into estrone (Simpson et al. 1994). In numerous fish, reptile, and amphibian species, cyp19 induction or inhibition produces female-biased or male-biased sex ratios, respectively (Chardard and Dournon 1999; Navarro-Martin et al. 2009; Richard-Mercier et al. 1995). Induction of *in vitro* cyp19 activity has been reported in human cell lines after exposure to ATZ (Heneweer et al. 2004; Holloway et al. 2008). However, several other studies have not observed such responses in amphibians (Coady et al. 2005; Hecker et al. 2005b; 2005a; Oka et al. 2008). The underlying reasons for these differences and the mechanism through which ATZ may disrupt vertebrate development remain unclear.

In the present study, we investigated alternative mechanisms through which ATZ may induce estrogen-like effects in amphibians. These mechanisms include the induction of estrogen receptor α (*eralpha*), which is activated upon estrogen binding and has been recognized as an estrogenic biomarker of estrogenic exposure (Lutz et al. 2005). Studies have shown that after treatment with estrogenic substances, *eralpha* expression increased in *Rana pipiens* tadpole brain [17α-ethinylestradiol (EE_2_); Duarte et al. 2006], the whole body of *Xenopus laevis* tadpoles (bisphenol A; Levy et al. 2004), and fish liver (EE_2_; Filby et al. 2007). The 5β-reductase (srd5beta) pathway is also potentially involved in the feminization of developing amphibians (Duarte-Guterman et al. 2010). A member of the aldo-keto reductase superfamily, srd5beta can regulate androgen bioavailability by catalyzing the conversion of testosterone to 5β-dihydrotestosterone (5β-DHT) reviewed by Langlois et al. (2009). Therefore, we hypothesized that exposure to ATZ alters *eralpha* mRNA level and srd5beta activity in the target tissues of exposed tadpoles.

There is also controversial evidence that ATZ affects amphibian development and metamorphosis (Coady et al. 2004; Freeman and Rayburn 2005). Several studies have reported developmental defects in amphibians after ATZ exposure (Brodeur et al. 2009; Coady et al. 2004; Freeman and Rayburn 2005; Larson et al. 1998; Lenkowski et al. 2008; Storrs and Kiesecker 2004). However, several other studies have not found any evidence that ATZ disrupts amphibian development even in the same species (Carr et al. 2003; Coady et al. 2005; Diana et al. 2000; Oka et al. 2008; Orton et al. 2006). These differential responses to ATZ exposure during amphibian development remain to be explained. In amphibians, metamorphosis is stimulated by environmental signals that impinge on the central control of the hypothalamus–pituitary–thyroid axis to initiate release of thyroid hormones [THs; thyroxine (T_4_), triidothyronine (T_3_)] into circulation. Conversion of T_4_ to T_3_ and subsequent degradation occur mainly in peripheral tissues and involve deiodinase enzymes (dio). THs then act through thyroid receptors (tr) that regulate gene expression by interacting with the thyroid response element in target genes (Aranda and Pascual 2001). Changes in the expression of *dio* and *tr* could influence the T_4_ to T_3_ ratio, which in turn will affect metamorphosis (Manzon and Denver 2004).

We investigated the effects of ATZ on sexual development and metamorphosis in *R. pipiens* under environmentally relevant conditions, as simulated in a mesocosm system. We assessed survival, success and age at metamorphosis, wet weight, snout–vent length (SVL), sex ratio, gonadal histology, gene expression (estrogen- and TH-responsive genes) and enzymatic activities (cyp19 and srd5beta). To our knowledge, this is the first study to use chronic ATZ exposure in amphibians and to evaluate changes in *a*) *cyp19* expression and activity simultaneously; *b*) *eralpha* expression; *c*) steroidogenic enzyme srd5beta activity; and *d*) *dio* and *tr* expression in premetamorphic and metamorphic tadpoles. Complementary field surveys were undertaken to confirm relevant environmental ATZ concentrations in water and the sex ratio in naturally metamorphosing tadpoles from the same population that we raised in captivity in the mesocosms.

## Materials and Methods

### Chemicals and reagents

The herbicide AAtrex Liquid 480 (Registration # 18450; purity 97.1% ATZ, 2.9% related triazines, and 5% ethylene glycol wt/vol; Syngenta Crop Protection Canada Inc., Guelph, Ontario, Canada) was purchased locally and used to mimic ATZ input into the environment. We purchased EE_2_ (CAS 57-63-6, purity ≥ 98%) from Sigma-Aldrich Canada Ltd. (Oakville, Ontario, Canada).

### Animals

Animals were collected in the Raisin River region (Cornwall, Ontario, Canada; latitude, N45°09′58.9″; longitude, W074°47′41.9″). For the field survey (summer 2006), young-of-the-year metamorphs were caught (*n* = 30) from our reference site [see Supplemental Material, Figure 1 (doi:10.1289/ehp.0901418)]. Animals were brought to the University of Ottawa on ice and anesthetized using a solution of 2% tricaine methanesulfonate (MS-222; Sigma-Aldrich Canada Ltd.). Animals were sacrificed by transection of the spinal cord. The kidney–gonad complex was removed and fixed in Cal-Ex II (Fisher Scientific, Ottawa, Ontario, Canada) for 48 hr and then transferred and preserved in 70% ethanol (EtOH) until histologic processing. During dissection, phenotypic sex was determined by visual inspection. For the mesocosm study, five fertilized egg masses were collected from the reference site. The egg masses were maintained in the laboratory in 10-L tanks of City of Ottawa water filtered with activated carbon (pH 6.6; dissolved oxygen, 8.4–10 mg/L; temperature, 20–21°C). After hatching, tadpoles were separated into extra tanks to allow a final density of approximately 1 g tadpole/L and were maintained on a 12-hr light/dark photoperiod. Water was aerated, and 5 L of the water was renewed twice weekly. Tadpoles were fed daily *ad libitum* with Nutrafin fish flakes (Hagen, Montreal, Québec, Canada) and frog brittle (Carolina Biological Supply Co., Burlington, NC, USA). The animals were kept in the laboratory until Gosner stage 25 (G25, beginning of independent feeding; Gosner 1960). Thirty tadpoles (six tadpoles from each of five different egg masses) were added to each of the mesocosms, for a final density of 0.1 g tadpole/L [American Society for Testing and Materials (ASTM) 2000]. All animal experimentation followed the guidelines and standards of the Animal Care Committee and the Canadian Council on Animal Care of the University of Ottawa. Animals were treated humanely and with regard for alleviation of suffering.

### Mesocosm conditions

We used an outdoor mesocosm design in this study to mimic natural pond settings. Each mesocosm consisted of a 378-L high-density polyethylene tank that was washed many times with pressurized water and aged for at least 2 years prior to the beginning of the exposure. In each mesocosm we added 300 L groundwater, 50 g rabbit pellets (Hagen), and 100 g dried leaves. We introduced *Daphnia magna* from a local creek to add food diversity in the mesocosms. Each mesocosm was constantly aerated using aquarium bubblers and covered with a lid of nylon shade cloth netting to exclude predators.

### ATZ exposure

Tadpoles at G27 were exposed to five nominal treatments: 0 μg/L ATZ (control; *n* = 5 mesocosms), 0.1 μg/L ATZ (*n* = 5), 1.8 μg/L ATZ (*n* = 5), 0.0003% EtOH (*n* = 5), or 1.5 μg/L EE_2_ dissolved in EtOH (0.0003%; *n* = 5). EE_2_ was used as a positive control for feminization because it induces intersex in *R. pipiens* (Hogan et al. 2008). Two ATZ applications were made on 12 May and 19 May 2006 to achieve nominal concentrations. Dissolved oxygen (milligrams per liter), pH, and temperature (°C) were recorded weekly. Ammonia levels, tested using the Lamotte Ammonia Kit (Lamotte, Chestertown, Maryland, USA), remained below the limit of detection (LOD, < 250 μg/L) throughout the experiment. Rain samples collected onsite were analyzed for ATZ content by high performance liquid chromatography with dual mass spectrometry (HPLC-MSMS), and concentrations were below the LOD (< 0.003 μg/L).

### Termination of the exposure

Animals reaching metamorphosis were removed at G42 (metamorphic climax). At the end of the exposure, from the remaining nonmetamorphosed tadpoles, we sampled a cohort of premetamorphic G34 tadpoles for gene expression analysis. We chose developmental stage G34 because tadpoles begin to respond to THs at this stage (Shi 1999). We anesthetized the animals by immersion in 1% MS-222 and recorded age at metamorphosis (AAM; days), wet weight (WW; grams), and SVL (millimeters). Animals were sacrificed by decapitation and brain, liver, and tail were removed. All samples were immediately frozen on dry ice and stored at −80°C. The kidney–gonad complex was also fixed for histology analysis.

### Water analysis

For the field survey, five different locations in the Raisin River (Ontario) were sampled every week. For the mesocosm study, water samples were collected regularly [see Supplemental Material, Figure 3 (doi:10.1289/ehp.0901418)]. Analysis of water samples was performed by Environment Canada for ATZ concentrations following the protocol of Hua et al. (2006). Water samples were spiked with 1 μg internal standard (ATZ-d_5_) and filtered on a 0.45-μm HATF membrane (Pall Life Science, VWR International, Mississauga, Ontario, Canada) before being concentrated on SPE LC-18 Supelclean cartridges (Supelclean, Sigma-Aldrich Canada Ltd.). ATZ was eluted from the SPE cartridges with methanol, and each sample was quantified using a calibration curve with six levels of standards ranging from 0.1 pg/μL to 10 pg/μL, with *r*^2^ > 0.99 using the internal standard method. The LOD for the HPLC-MSMS was 0.003 μg/L.

### Sex ratio and histology

Upon dissection, all animals were classified as either male or female by visual inspection of gonadal gross morphology using a dissecting microscope (4×). We randomly chose a subset of male samples for histologic analysis to determine the presence of testicular oocytes. Fixed gonads were embedded in paraffin, serially sectioned longitudinally at 5-μm intervals, and stained with hematoxylin and eosin. A blind analysis was performed.

### Real-time reverse transcriptase-polymerase chain reaction (RT-PCR)

Samples were homogenized at 20 Hz for 2 min. Total RNA from whole brain and liver tissues (from G34 and G42 animals) was isolated using the QIAGEN RNeasy Micro Kit and RNeasy Mini Kit (Qiagen, Mississauga, Ontario, Canada), respectively. TRIzol reagent (Invitrogen, Canada Inc., Burlington, Ontario, Canada) was used to isolate total RNA from tadpole tails for both stages. RNA was resuspended in RNase-free water and stored at −80°C. Concentrations of RNA were determined using GeneQuant RNA/DNA calculator (Amersham Pharmacia Biotech, Piscataway, NJ, USA). Total cDNA was prepared from 1 μg and 2 μg (from G34 and G42 animal tissues, respectively) of total RNA and 0.2 μg random hexamer primers (Invitrogen) using Superscript II reverse transcriptase (Invitrogen). All procedures followed manufacturer protocols.

We used real-time RT-PCR simplex (SYBR Green detection) and multiplex assays (dual-labeled fluorescent probes), as described by Hogan et al. (2007), to detect transcripts for *cyp19, eralpha*, TH receptor isoforms (*tralpha* and *trbeta*), deiodinases 2 and 3 (*dio2* and *dio3*, respectively), and the ribosomal protein L8 (*rpl8*). The stress neuropeptide corticotropin-releasing hormone (*crh*) was also analyzed by RT-PCR in tadpole brains as described by Croteau (2009). Samples were amplified in duplicate along with negative controls (no template and no reverse-transcriptase controls). Each reaction exhibited an efficiency of 100% ± 10%, with *r*^2^ > 0.985. Data were normalized to *rpl8* mRNA and are presented as fold-change relative to controls.

### Enzyme activity analyses

We determined enzymatic activity of cyp19 and srd5beta using radiometric methods according to Langlois et al. (2010). The tritiated water method was used to assess cyp19 activity in pools of two brains from G42 animals of the same sex, and cyp19 activity is expressed as femtomoles ^3^H_2_O per hour × milligrams of protein. The activity of srd5beta was determined by the conversion of ^14^C-testosterone into ^14^C-5β-reduced metabolites (5β-DHT and 5β-androstan-3β-17β-diol) in individual tadpole livers at G42 and is expressed as the sum of 5β-DHT and 5β-androstan-3β-17β-diol per hour × milligrams of protein. We measured total protein concentration using the Bio-Rad Protein Assay kit (Bio-Rad, Hercules, CA, USA).

### Statistical analysis

We used Pearson’s chi-square test to determine statistical differences for sex ratio, survival, and success of metamorphosis. We used one-way analysis of variance (ANOVA) to analyze WW, SVL, AAM, and G34 tadpole gene expression data and two-way ANOVA to analyze G42 tadpole gene expression and enzymatic activity data. ANOVAs were followed by Bonferroni post hoc test (when warranted). Data were tested for normality (Kolmogorov-Smirnov test) and homoscedasticity (Levene’s test). When data failed to meet the assumptions after transformation, we used the nonparametric Kruskal-Wallis one-way ANOVA on ranks, followed by the Mann-Whitney *U* test. ATZ and EtOH treatment data were compared with water control, and EE_2_ treatment data were compared with the EtOH solvent control.

## Results

### ATZ concentrations and mesocosm parameters

ATZ was detected in every stream sampled on the Raisin River [see Supplemental Material, Figure 1A (doi:10.1289/ehp.0901418)], and concentrations ranged from 0.01 to 1.6 μg/L (see Supplemental Material, Figure 1B). ATZ concentrations in the 0.1-μg and 1.8-μg ATZ treatment groups were 0.09–0.21 μg/L (nominal 0.1 μg/L ATZ) and 1.6–3.7 μg/L (nominal 1.8 μg/L ATZ), respectively; for details, see Supplemental Material, Figure 2. ATZ concentrations in the control ranged from the LOD (0.003 μg/L) to 0.028 μg/L ATZ (for details, see Supplemental Material, Figure 2). To compensate for water loss through evaporation, groundwater was added regularly to the mesocosm. A small input of ATZ to groundwater coming from peripheral agricultural fields after the spraying season could explain detectable ATZ in the water control on 9 June 2006; however, all control replicates were < 0.008 μg/L ATZ on 21 July 2006. We found no statistical differences in pH, dissolved oxygen, or temperature measurements among tanks for every monitored event (*p >* 0.05; Supplemental Material, Figure 3). Furthermore, at pretreatment, the physicochemical parameters did not vary among treatments and averaged 6.2 mg/L dissolved oxygen and 17.9°C. Results are reported using the nominal concentrations to facilitate presentation.

### ATZ affects metamorphic success

High survival rates occurred in water (79%) and EtOH (76%) controls (Table 1). The survival rate in the 0.1-μg/L ATZ group was 75%, which was not different from control. However, the 66% survival rate in the 1.8-μg/L ATZ group was significantly lower than control (*p* < 0.05). The EE_2_ group also exhibited a significant decrease in survival rate (65%) compared with its EtOH control (76%; *p* < 0.05). Significantly fewer ATZ- and EE_2_-treated tadpoles reached metamorphosis (data were corrected for mortality).In the controls, 76% water control and 85% EtOH control tadpoles reached metamorphosis, whereas 45%, 50%, and 55% completed metamorphosis in 0.1 μg/L ATZ; 1.8 μg/L ATZ, and EE_2_ treatments, respectively (*p* < 0.001). We found no significant effects of treatment on AAM, SVL, and WW, except that EE_2_-exposed animals were 5.6% smaller in length and 18% lower in weight, on average, than the EtOH control (*p* < 0.05). There was no effect of treatment on brain *crh* mRNA levels (data not shown).

### ATZ induces female-biased sex ratio

The sex ratios of surviving metamorphs for control groups were 1:0.6 (male:female) and 1:0.6 in the water and EtOH controls, respectively (Table 2). These ratios are comparable with wild-caught metamorphosing animals (1:0.5) from our reference site where we collected eggs for the mesocosm experiment that year. Only the highest ATZ exposure (1.8 μg/L ATZ) significantly altered sex ratio to 1:1.4 (*p* < 0.01), whereas the 0.1 μg/L ATZ treatment yielded a sex ratio similar to the water control (1:0.8). Exposure to EE_2_ did not change the sex ratio (1:0.9); however, histologic analysis indicates that 22% of EE_2_-treated animals expressed an intersex condition (Table 2). In contrast, we found no intersex gonads in field-collected, water control, EtOH control, or ATZ-treated animals.

### ATZ did not induce *cyp19* gene expression and activity

**Figure 1:**
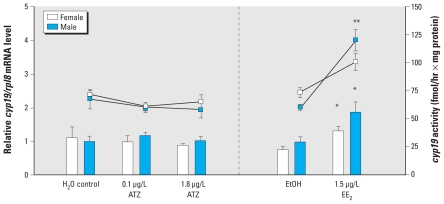
Effect of chronic ATZ and EE_2_ exposures on brain *cyp19* expression and enzyme activity in *R. pipiens* (G42 metamorphs) as determined by real-time RT-PCR. The levels of *cyp19* mRNA are expressed relative to the water control group and are normalized to the expression of *rpl8* (bars; left *y*-axis). The activity of cyp19 was assessed using radiometric method and is expressed in fmoles/hr normalized to protein content (lines; right *y*-axis). Values represent mean ± SE (*n* = 6–8). *p ≤ 0.05 for mRNA differences, and **p ≤ 0.05 for activity differences, compared with controls by two-way ANOVA followed by Bonferroni post hoc comparisons.

ATZ did not significantly affect *cyp19* mRNA level or activity in the brain of G42 animals (Figure 1). In contrast, EE_2_ significantly increased both *cyp19* mRNA level (*p* < 0.01) and activity (*p* < 0.001) in the brains of G42 females and males. No changes were observed in *cyp19* mRNA levels in the brains of the G34 tadpoles (data not shown).

### Effects of ATZ on gene expression

**Figure 2:**
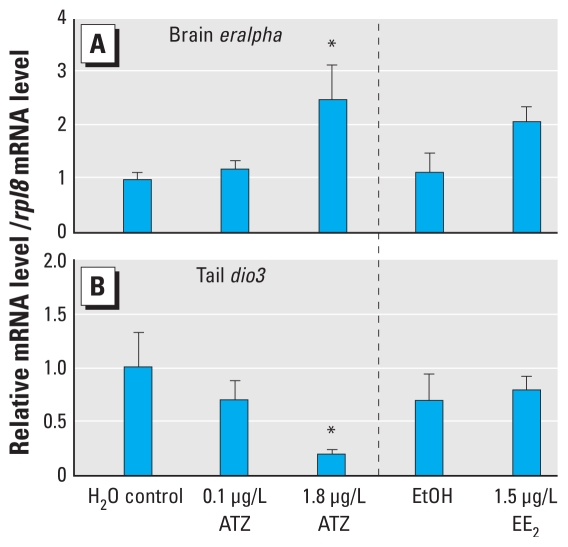
Effect of chronic ATZ and EE_2_ exposures on the expression of brain *eralpha* (*A*) and tail *dio3* (*B*) in *R. pipiens* determined by real-time RT-PCR on premetamorphic G34 tadpoles that failed to metamorphose. The mRNA levels are expressed relative to the water control group (0 μg/L ATZ) and are normalized to the expression of *rpl8*. Values represent the mean + SE (*n* = 8). *p ≤ 0.05 compared with controls by one-way ANOVA followed by Bonferroni post hoc comparisons.

The expression of *eralpha* in the brains of G34 tadpoles in the 1.8 μg/L ATZ group was 2.5 times that of the water control (*p* < 0.01; Figure 2A). We observed no changes in expression of *eralpha* mRNA in the brains of G42 tadpoles or in the livers from G34 and G42 tadpoles (data not shown). EE_2_ did not significantly affect the expression of *eralpha* mRNA in any tissue regardless of sex or stage. To determine if the lower metamorphic success observed after ATZ exposure was accompanied by disruption of the thyroid axis, we measured thyroid-related gene expression. We detected a decrease in *dio3* mRNA in tail tissue of G34 animals exposed to 1.8 μg/L ATZ (*p* < 0.05; Figure 2B). No other *dio3* mRNA changes were identified in brain and liver of G34 and G42 animals, and no changes were detected for *dio2*, *tralpha,* or *trbeta* expression in brain, liver, or tail for both stages (data not shown).

### ATZ affected a sex difference in srd5beta activity

**Figure 3:**
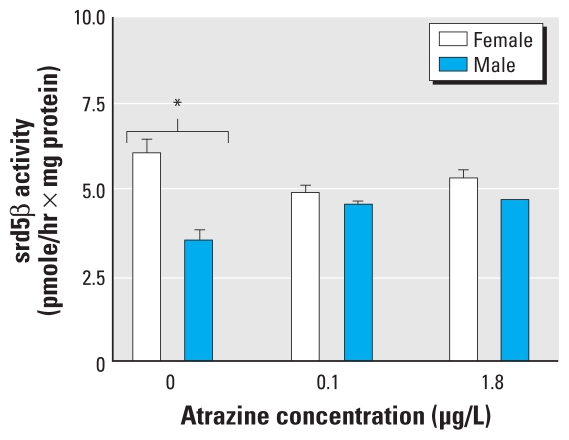
Effects of chronic ATZ exposure on liver srd5beta activity in *R. pipiens* determined at G42 using the radiometric method and expressed in pmol/hr (srd5beta) normalized to protein content. Values represent the mean ± SE (*n* = 6). *p ≤ 0.05 by two-way ANOVA followed by Bonferroni post hoc comparisons.

We identified a conspicuous sexual dimorphism in srd5beta activity in animals in the water control group, in which the livers of the females expressed 43% more activity than the males (*p* < 0.001; Figure 3). Interestingly, this sex difference was abolished in animals exposed to either ATZ treatment *(p* < 0.01).

## Discussion

This study supports the theory that environmentally relevant ATZ exposure in outdoor mesocosms affects both amphibian gonadal differentiation and metamorphosis. We found evidence that ATZ may feminize *R. pipiens* when tadpoles are exposed chronically to low concentrations of ATZ, and we suggest that associated changes in brain *eralpha* mRNA level and liver srd5beta activity might be involved in producing the response. We also observed that ATZ affects metamorphosis by decreasing the numbers of frogs reaching metamorphosis. In addition, we investigated for the first time the effects of ATZ on thyroid-related gene expression in amphibians.

In our experiments, ATZ exposures led to a female-biased sex ratio, as the nominal 1.8 μg/L ATZ treatment produced 20% more female phenotype animals compared with animals reared in untreated water. A recent study (Oka et al. 2008) demonstrated that wild-type male *X. laevis* tadpoles exposed to 0.1–100 μg/L ATZ also displayed a dose-dependent increase in female phenotype. Conversely, other studies reported no bias in *X. laevis* sex ratio after chronic ATZ treatments (Carr et al. 2003; Kloas et al. 2009). In addition to female-biased sex ratio, studies have shown that ATZ increases the incidence of intersex condition in amphibians [*X. laevis* (Hayes et al. 2002a), *R. pipiens* (Hayes et al. 2003)]. However, we did not observe intersex gonads in ATZ-exposed *R. pipiens.* These differences between studies may be associated with differences in experimental designs (e.g., different species, stages at exposure, duration of exposure, and other exposure conditions). In contrast, 22% of our EE_2_-treated male tadpoles displayed testicular oocytes. These results from outdoor mesocosm exposures confirmed a previous laboratory study in which chronic exposure of *R. pipiens* tadpoles to 1.5 μg/L EE_2_ in a static renewal system resulted in 30% of *R. pipiens* metamorphs exhibiting an intersex condition (Hogan et al. 2008). Thus, the experimental mesocosm design functioned successfully as an exposure system, and our population of *R. pipiens* has the capacity to respond to estrogenic compounds.

Many attempts have been made to investigate possible estrogenic mechanisms of ATZ action in several vertebrate models. ATZ failed to induce estrogen-mediated responses in the uterus of immature female Sprague-Dawley rat, in the estrogen-responsive MCF-7 human breast cancer cell line, and in the estrogen-dependent recombinant yeast strain PL3 (Connor et al. 1996). Moreover, ATZ also failed to induce vitellogenin production *in vivo* in *X. laevis* liver and *in vitro* in *X. laevis* hepatocyte cultures after exposures to levels ranging from 0.1 to 100 μg/L ATZ (Oka et al. 2008). Because ovarian differentiation in amphibians is mediated by estrogens, the dominant hypothesis in the literature remains that ATZ induces cyp19 activity (Hayes et al. 2002a, 2003; Tavera-Mendoza et al. 2002a, 2002b). There is evidence that cyp19 activity is induced indirectly through phosphodiesterase inhibition (Roberge et al. 2004) and through binding to steroidogenic factor 1 (Fan et al. 2007). Whether induction of cyp19 activity is the only estrogenic action of ATZ is still a matter of debate. Several studies have refuted the cyp19 induction hypothesis in amphibians (Coady et al. 2005; Hecker et al. 2005a, 2005b; Oka et al. 2008). Our data also support that ATZ action is not mediated via cyp19 activity induction, because we detected no changes in *cyp19* mRNA level or a cyp19 enzyme activity in *R. pipiens* tadpole brain. Taken together, the cyp19 hypothesis for ATZ disruption of sexual development is not well supported; therefore, we investigated other potential mechanisms of action.

We studied *eralpha* expression and srd5beta activity, two pathways that, if altered by ATZ, could produce an estrogen-like response. Our data confirmed that tadpoles exposed to 1.8 μg/L ATZ at G34 expressed higher *eralpha* mRNA levels in brain compared with control animals. Similar increases in *eralpha* expression in *R. pipiens* have been reported after EE_2_ treatment under laboratory conditions (Duarte et al. 2006). In our mesocosm study, EE_2_ doubled the brain *eralpha* mRNA level. This suggests that estrogenic compounds have the ability to increase *eralpha* mRNA levels. Hogan et al. (2007) reported that expression of brain *eralpha* mRNA was similar at G30 and G36, a period that coincides with gonadal differentiation. It is therefore possible that a 2.5-fold increase in *eralpha* expression at G34 after ATZ exposure could alter the sensitivity of the developing brain to estrogen and lead to other physiologic changes. *R. pipiens* tadpoles are most sensitive to EE_2_-induced feminization early in development, before G30 (Hogan et al. 2008); therefore, amphibians exposed to ATZ in early development may be more sensitive to ATZ-induced feminization.

We also demonstrated that ATZ alters hepatic srd5beta activity in *R. pipiens* tadpoles. Recent studies in our laboratory have shown that inhibiting srd5beta activity resulted in a female-skewed sex ratio, suggesting that this enzyme could be involved in amphibian gonadal development (Duarte-Guterman et al. 2010). In mammals, a natural sexual dimorphism exists in hepatic srd5beta activity; for example, female rat liver contains more srd5beta activity than male liver (Cooke GM, unpublished data). In the present study, we observed a similar dimorphic pattern in the livers of H_2_O-control tadpoles. However, after exposure to ATZ, this sex difference in srd5beta activity disappeared. There is a lack of data regarding the importance of this sexual dimorphism, but it likely results in differential androgen status in developing males versus females. In addition to a possible role in gonadal development, srd5beta is also involved in other biological functions such as erythropoiesis (Garavini and Cristofori 1984) and bile biosynthesis (Kondo et al. 1994); thus, srd5beta alteration could also lead to other physiologic defects. Future studies should explore these new end points for ATZ action.

In amphibians, THs are essential for metamorphosis and are involved in the remodeling of TH target tissues such as brain, hindlimb, intestine, and tail (Shi 1999). A disruption in TH production can result in important physiologic defects. Developmental exposure of *X. laevis* to ammonium perchlorate (an inhibitor of thyroidal iodide uptake) resulted in fewer tadpoles completing tail resorption, forelimb emergence, and hindlimb development (Goleman et al. 2002). Several studies have suggested that ATZ alters the thyroid axis in *X. laevis* (Freeman and Rayburn 2005) and rats [female albino rats (Kornilovskaya et al. 1996); male Wistar rats; (Stoker et al. 2000)]. Here, we present evidence that ATZ exposure alters the thyroid axis by affecting success of metamorphosis and also TH-related gene expression in *R. pipiens*. Our real-time RT-PCR results indicate a 79% reduction of *dio3* mRNA in G34 tadpole tails. This decrease in mRNA level is most likely caused by a compensatory mechanism of the animals to trigger metamorphosis by reducing T_3_ breakdown to inactive metabolites.

## Conclusions

Using an outdoor mesocosm design, we found that ATZ can affect amphibian development at levels measured in water across the distribution of *R. pipiens* in North America (Graymore et al. 2001). Much controversy surrounds the effects of ATZ on frogs. For example, at one extreme, *X. laevis* exposed to low levels of ATZ under laboratory conditions suffered gonadal dysgenesis (Hayes et al. 2006), whereas at the other extreme, no effects were observed after similar exposures (Coady et al. 2005; Kloas et al. 2009). The reasons for such differences are numerous and have been discussed previously (Hayes 2004; Solomon et al. 2008), and there is the question of the relevance of studies with nonnative species to predict potential effects on indigenous species. We used a mesocosm study to directly test the effects of a commercial ATZ preparation on a North American native species. Our results using mesocosms are somewhat intermediate compared with previous laboratory and field studies. Nevertheless, the present study demonstrates that ATZ can be biologically active in *R. pipiens*, as we report female- biased sex ratios and disruption of metamorphosis with associated changes in gene expression after ATZ exposure. These responses occurred with environmentally relevant exposure conditions. Female-biased sex ratio and disruption of metamorphosis are important physiologic consequences of this exposure, which could potentially alter amphibian population fitness. Therefore, subsequent studies should examine population-level effects associated with the widespread use of ATZ with particular focus on risks to native amphibian populations.

## Figures and Tables

**Table 1:** Effects of ATZ and EE_2_ on *R. pipiens* development and metamorphosis.

Treatment	*n*	Survival (*n*)^a^	Metamorphic success (*n*)^a^	AAM (days)^b^,^c^	SVL (mm)^b^,^c^	WW (g)^b^,^c^
H_2_O control	150	118	90	71.5 ± 1.5	17.9 ± 0.3	0.96 ± 0.06
0.1 μg/L ATZ	150	113	50*	78.8 ± 2.9	17.4 ± 0.1	0.89 ± 0.03
1.8 μg/L ATZ	150	99*	47*	74.9 ± 4.0	17.8 ± 0.5	0.98 ± 0.06
EtOH	150	114	99	76.1 ± 3.6	17.9 ± 0.4	1.01 ± 0.04
1.5 μg/L EE_2_	150	97**	52**	75.8 ± 4.3	16.9 ± 0.4**	0.83 ± 0.05**

a Includes all animals that reached or passed G42.

b Includes only the animals at G42.

c Mean ± SE.

* *p* ≤ 0.05 compared with the water control, and

** *p* ≤ 0.05 compared with the EtOH control, using either the chi-square test or the one-way ANOVA test.

**Table 2:** Gonadal gross morphology and histologic analysis of *R. pipiens* from the mesocosms and from the Raisin River.

		Gonadal gross morphology			Gonadal histology	
Treatment	*n*^a^	Male	Female	Sex ratio (M:F)	Male^b^(*n*)	TO (%)
H_2_O control	60	37	23	1:0.6	20	0
0.1 μg/L ATZ	34	19	15	1:0.8	17	0
1.8 μg/L ATZ	31	13	18	1:1.4*	12	0
EtOH	66	42	24	1:0.6	17	0
1.5 μg/L EE_2_	35	18	17	1:0.9	18	22**
RR reference site	30	20	10	1:0.5	10	0

Abbreviations: F, female; M, male; RR, Raisin River; TO, testicular oocytes. Data represent sample size (*n*) and sex ratio (male to female ratio) of G42 frogs in all five treatments and in wild-caught metamorphs from reference site R [see Supplemental Material, Figure 1 (doi:10.1289/ehp.0901418)].

a From the metamorphosed animals, only G42 individuals were used for gonadal gross morphology.

b Randomized sub-samples of males were chosen for gonadal histology.

* *p* ≤ 0.05 compared with the 0 μg/L ATZ control, and

** *p* ≤ 0.05 compared with the EtOH control, using the chi-square test.
